# New Appliances and Things Medical

**Published:** 1906-08-04

**Authors:** 


### NEW APPLIANCES AND THINGS MEDICAL.

[We shall be glad to receive, at our Office, 28 & 29 Southampton Street, Strand, London, W.C., from the manufacturers, specimens of all new preparations and appliances which may be brought out from time to time.]

### THE CAMBRIDGE LEMONADE.

(Chivers and Sons, Limited, Histon, Cambridge.)

The sample before us consists of a powder carefully prepared from Sicilian lemons and free from any added foreign ingredient. In order to convert it into lemonade all that is required is boiling water and three quarters of a pound of loaf sugar. In the first instance, one pint of boiling water should be used, and the resulting syrup can be kept for a considerable time in a stoppered decanter, and may be subsequently diluted with water or mineral water to taste. It is estimated that a 4½d. bottle of this lemonade powder will make two gallons of lemonade. At this time of year the value of a cool and pleasant diluent drink is very obvious to all of us, and we have no doubt whatever in expressing the opinion that by putting this lemonade on the market Messrs. Chivers have supplied a very great demand. We can strongly advise medical men to recommend it to their patients as a most inexpensive and pleasant summer beverage.

